# Iodine deficiency among goiter patients in rural South Sudan

**DOI:** 10.1186/1756-0500-7-751

**Published:** 2014-10-23

**Authors:** Chep C Chuot, Moses Galukande, Charles Ibingira, Nicholas Kisa, Jane Odubu Fualal

**Affiliations:** Surgery Department, College of Health Sciences, Makerere University, Kampala, Uganda; Anatomy Department, College of Health Sciences, Makerere University, Kampala, Uganda

**Keywords:** Goiter, Iodine, Deficiency, Rural south Sudan

## Abstract

**Background:**

It is estimated that 2.2 billion or approximately 30% of the world’s population live in iodine-deficient areas. In a 2005 study households consuming iodized salt in South Sudan increased from 40% to 73%. Despite this achievement, there are still many goiter cases in rural South Sudan and iodine deficiency remains as a major public health problem in this part of sub Saharan Africa.

The purpose of this study therefore was to determine the prevalence of iodine deficiency among rural Southern Sudan goiter patients.

**Methods:**

A cross-sectional study was carried out in three South Sudan counties, adults with goiter were from three centers following a mobilization campaign that lasted 4 weeks for free medical care. They were clinically evaluated and completed interviewer administered questionnaires to determine their age, gender, diet, family history, drug history, and medical history. Urine samples were then taken for urinary iodine levels. The outcome was iodine deficiency measured as urinary iodine less than 100 μg per/ L. Multiple logistic regression was used to establish the factors associated with iodine deficiency in South Sudan. Ethical approval was obtained.

**Results:**

A total of 286 goitre patients were recruited. The mean age was 38 years (SD 9), 262(92%) were females (F: M ratio 11:1), and 257(90%) were rural- peasants, 25% (20/286) had moderate to severe iodine deficiency. 174(62%) consumed non-iodized salts.

**Conclusion:**

Iodine deficiency is highly prevalent among rural South Sudan communities and a likely cause for goiters. Rural poor women are highly vulnerable.

## Background

It was estimated that over 2.2 billion participants worldwide are at risk of iodine deficiency. Iodine deficiency remains a major public health problem [1, 2]. Understanding the role of risk factors is key to developing a clear and effective strategy for improving global health. In low-income countries relatively few risk factors such as micronutrient deficiency are responsible for a large percentage of and ill health [3]. The mandatory use of iodized salt reduces or eliminates iodine deficiency [3]. In South Sudan according to Gaffar and Mahafuz, in a 2005 survey, households consuming iodized salt had increased from 40 to 73% [4]. Although the International Council for Control of iodine deficiency disorders (ICCID) indicates that South Sudan, Sudan and Ethiopia have only 35% or less households with access to iodized salt, neighboring Kenya and Uganda records 90% and more [3, 5–7]. However, high goiters prevalence persist in these high coverage iodized salt intake regions. Severe iodine deficiency results in impaired thyroid hormone synthesis and or/thyroid enlargement (goiter). Population effects of severe iodine deficiency, termed iodine deficiency disorders (IDDs), include endemic goiter, hypothyroidism, cretinism, decreased fertility rate, increased infant mortality and mental retardation [8]. There is no data from south Sudan describing the association of goiter and iodine deficiency.

The purpose of this study was to determine the prevalence iodine deficiency among adult patients with goiter and associated factors in Unity state of south Sudan.

### Country context

The Health Care System in Sudan is currently described as critical, the country’s most important industry oil, which is a source of 98% of the country’s revenue suffers frequent shut downs due to insecurity and political upheaval. There is an enormous, largely unmet demand for health services including immediate humanitarian crises often involving internally displaced persons. There are inadequate human resources for health, infrastructure and other resources to meet current needs [9].

## Method

### Study design

A cross-sectional descriptive community based study to determine the prevalence of iodine deficiency.

### Study setting

South Sudan; involving three counties: Rubkona county with a population of 100,236 has 170 villages health facilities in an area of 3,368 km^2^, its major livelihood activities are farming, live stock and fishing. Koch county with a population of 74,863, Guit county with a population of 33,004 [3, 10].

These three counties were purposively selected because they have a stable population with no much people migration as a result of better security and civil order compared to the other 6 counties in the country. No established patient records exist in these counties to give a sense of the extent of the problem.

The population in this area is socio economically homogenous, its rural and engage in subsistence activities and the majority reside along the river Nile where floods frequently occur.

### Study population

All Adults (both males and females) aged 18 years and above residing in Rubkona, Guit and Koch counties of the Republic of south Sudan with a visibly enlarged thyroid gland were enrolled.

### Sampling procedure

Each county had health centers to which study participants presented. Three health centers from Rubkona County, two from Guit, and one from Koch County were selected randomly out of the nine as study sites. The number of centers selected from each County was determined by probability proportionate to size of the population of the three counties.

### Data collection

Local radio announcements were made a week prior to starting the study to encourage and alert the communities about the availability of free neck medical examinations, and testing.

The persons who responded to the invitation and were above 18 years and provided written informed consent, were examined. The physical examination for the presence of goiter was by inspection (visible goiter) and palpation. The simplified classification of goiter by World Health Organization (WHO) [11] was used as Grade 0, 1 and 2. Grade 1: A goiter is palpable but not visible where the neck is in the normal position. A thyroid gland was considered goitrous when each lateral lobe had a volume greater than the terminal phalanx of the thumbs of the subjects examined. The examinations were performed by trained research assistants (clinical officers and medical officers).

Grade 2: A swelling in the neck that is clearly visible when the neck is in a normal position and is consistent with an enlarged thyroid when the neck is palpable. Unique codes were assigned to each participant to ensure confidentiality. A questionnaire was completed and two fresh on-spot urine samples was collected from the participants as they came in and examined for iodine concentration and another sample was sent to another laboratory for quality control assurance. The Sandell-kolthoff method was used [12] which depends on iodine’s role as a catalyst in the reduction of cericammonium sulfate (yellow color) to the cerous form (colorless) in the presence of arsenious acid. Results were interpreted in accordance to WHO classification [13]. Optimal levels ranges 100-199 mcg/l, mild deficiency ranges 50–99 mcg/l, Moderate ranges 20–24 mcg/l, severe ranges <20 mcg/l, more than adequate rages 200–299 mcg/l, and excessive >299 mcg/l.

### Study variables

Socio demographic data included; age, gender, family history of goiter, dietary intake of iodized salt, food with goitrogens, medications, and levels of urinary iodine concentration (UIC) measure and goiter.

### Data analysis

Continuous data were summarized into mean and standard deviations and medians. Other categorical data were summarized as frequencies and percentages. The prevalence of iodine deficiency was the number of patients with goiter who had urine iodine below 100 mcg/l to the total number of goiter patients sampled for urine iodine concentration.

### Ethical considerations

Ethical approval was obtained from Makerere College of Health Sciences, School of Medicine Research and Ethics Committee, and the Ugandan National Council of Science and Technology and the Ministry of Health, South Sudan.

All participants provided informed written consent.

## Results

The study was conducted in the three counties of the Unity State in South Sudan between April and June 2012. A total of 286 patients with endemic goiter were interviewed and urinary iodine excretion was assessed. The mean age was 38 years (SD 9) with median age of 38 years (see Table 1).

**Table 1:** **Clinical features and socio- demographic profile of the respondents**

Variable	Category	Frequency	Percentage (%)
**County (Payam)**	Rubkona	179	63
	Guit	68	24
	Koch	39	14
**Gender**	Male	24	8
	Female	262	92
**Occupation**	*Peasants	257	90
	^†^Salaried/wage workers	30	11
	^∞^Business	1	0.4
**Symptoms of presenting goiter**			
	Neck swelling	285	99
	Palpitation	3	1
	Anxiety	4	1
	Difficulty in breathing	2	0.7
	Voice change	1	0.3
	Temperature intolerance	2	0.7
	Profuse sweating	3	1.0
**Signs of goiter**			
	Thyroidectomy scar	1	0.4
	Tenderness of mass	4	3
	Hoarseness of voice	1	0.7
	Grade 1 goiter	156	54.5
	Grade 2 goiter	130	45.5

*Subsistence farmers ^†^Low income/salaried workers ^∞^Business.

Of all the 286 participants, 262 (92%) were females, and 257 (90%) were peasants. (179) 63% were from Rubkona with 68 (24%) and 39 (14%) from Guit and Koch Payams respectively. The median duration of stay at the respective addresses was 36 years (25–42), 255 participants (90%) were indigenous (born within that area). Those who came from other places had stayed for median duration of 21 years (11–29). A handful (30 participants (11%) came from the Khartoum, Durfur, Bar el gazel and Malakal states. All respondents presented with a neck swelling, followed by anxiety 4 (2%), palpitations and profuse sweating each at 3 (1.0%). Voice change was seen in one respondent (0.3%). 156 (55%) had grade 1 goiter, 130 (46%) had grade 2 goiter, 1 (0.4%) had a thyroidectomy scar, 1 (0.7%) had hoarseness of voice, and tenderness of the swelling 4 (3%) see Table 2. The median urinary iodine secretion of participants with goiter was 152 μg/ml (IQ 101, 197) with mean of 151 and a standard deviation of 72 μg/ml. The median was preferred because the data on the urinary iodine secretion was not normally distributed with the majority of the participants skewed to the higher levels. See Table 3.

**Table 2:** **Factors associated with goiter in unity state Southern Sudan, 2012**

Variable	Category	Frequency	Percent (%)
Thyroid status	Nodular	279	98
	Multinodular	7	2
Previous surgery	Yes	1	1
	No	285	99
Present treatment	None	274	96
	Anti thyroid drugs	6	2
	Iodine	3	1
Sorghum/Maize	Yes	286	100
Iodized salt ingestion	Yes	108	38
	No	174	62
*Ingested iodized salt and had iodine deficiency	-	25	9
^†^Did not ingest iodized salt and had iodine deficiency	-	46	16
Family history of goitre	Yes	6	2
	No	276	97

*2 were moderate and 23 were severe.

^†^38 were moderate and 8 were severe.

**Table 3:** **Factors associated with goiter (urinary iodine levels as a proxy variable)**

Variable	F test	P value
Age	0.444	0.506
Sex	0.046	0.830
County	0.224	0.800
Duration of stay	0.973	0.325
Family history	1.425	0.234
Community history of goiter	1.845	1.176
Ingestion of iodized salt	0.131	0.878
**Diet**		
Maize	0.474	0.492
Sorghum	1.445	0.230
Previous history of thyroidectomy	0.169	0.681
Metabolic status of the thyroid	0.313	0.575

Table 4 indicates the magnitude of the problem 75% of the respondents had optimal to excess levels iodine excretion in their urine, deficiency was observed in 25% of the respondents while 7% had moderate to severe iodine deficiency levels. Of the 286 respondents, 279 (98%) had nodular goiter, 274 (96%) had never received any treatment, only one was had prior thyroidectomy done. All the respondents reported sorghum and maize as their predominant food, and 108 (38%) had been ingesting iodized salt. Family history of goiter was reported among 6(2%) of the respondents.

**Table 4:** **Comparison of the respondents’ results with WHO classification of iodine deficiency in Unity State**

WHO Classification	Cut offs	Frequency	Percent (%)
Severe deficiency	<20	11	4
Moderate deficiency	20 - 49	9	3
Mild deficiency	50 - 99	51	18
Optimal	100 - 199	149	52
More than adequate	200 - 299	58	20
Possibly excess	>299	8	3

Table 5 shows the comparison of variables among those that consumed iodized salt and those that did not.

**Table 5:** **Comparing variables of those who consumed iodized salts vs those that did not consume iodized salt**

Variable	Those who consumed iodized salts	Those that did not consume iodized salt
Gender		
Male	10	14
Positive family history of goiter	2	3
Iodine deficiency– Severe	23	8
Iodine deficiency– Moderate	2	38
Iodine deficiency – Mild	0	0
More than adequate	18	37
Optimal iodine	62	86
Excess iodine	3	5

The correlation between goiter size and urinary iodine levels Chi- square 4.461 p = 0.813.

**Figure 1:**
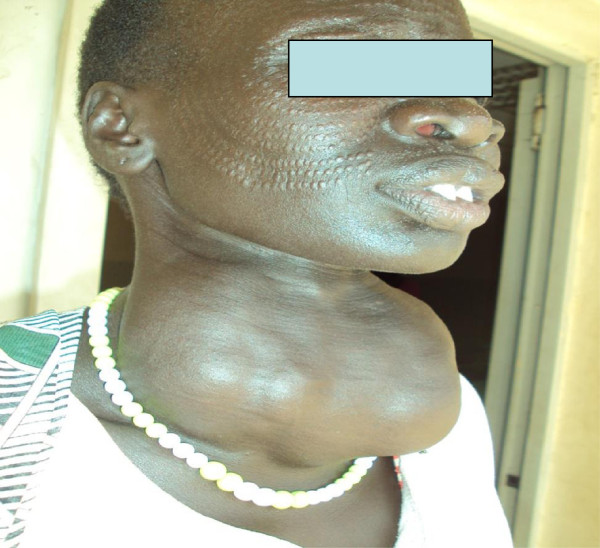
**Illustration of a participant with a goiter in South Sudan, 2012.**

274 (96%) had never received any treatment for the goiter.In Figure 1, a participant with a goiter is shown.

## Discussion

We set out to investigate the prevalence and factors associated with iodine deficiency among goiter patients in rural south Sudan. We found that patients with goiters were predominantly female (ratio 11:1). They had this thyroid condition nearly all their lives (mean age of 38 years and duration of symptoms was 35 years). Only 9 out of 286 the participants had received any form of medical attention. This attests to the degree of limitation of access to health care. They were mostly the rural poor, only 38% had access to iodized salt, contrary to Gaffar findings in 2005 [4] which suggested that households consuming iodized salt in south Sudan had increased from 50% to 70%. However the UIC data in this study indicates that 52% had optimum iodine levels and 25% clearly deficient. These data therefore suggests that nearly half of the study population had sub optimal iodine levels and therefore at risk of thyroid dysfunction. Some neighboring countries in the region such as Uganda and Kenya have over 90% of their populations consuming iodized salt [14] and it is therefore anticipated that iodine deficiency associated disorders are minimized which would include cretinism among children, goiter formation and hypo or hyper thyroidism.

What is also clear from these data is that, some individuals that ingested iodized salt were found to be iodine deficient. Only half of the individuals that did not ingest iodized salt were iodine deficient. Among the reasons for this apparent discrepancy is the fact that deficiency of iodine may be influenced by differences in diet.

Assessment of progress in controlling iodine deficiency is shifting from reliance on physical assessment of goiter to biochemistry, using urinary iodine concentrations. While the latter is less subjective, it measures current iodine states (over the last few days before sampling) whereas a physical goiter reflects a considerably longer history. None the less the goiter prevalence does respond and reductions can be observed at least from year to year with increased iodine intakes [15–17]. Trends in goiter prevalence from available repeated national surveys from 31 countries show a consistent picture of almost universal substantial improvement often from 30% to 40% down to single digit percentages over 10 – 20 years [14].

Most respondents had stayed with goiters for over 35 years, giving a sense of endemicity of iodine deficiency and limited access to health care. Universal iodized salt program was only introduced in 2005 by the Unity State government [18].

Over 75% had sufficient iodine using urinary iodine as a marker, the presence of goitres precedes the USI program. Perhaps what we are seeing is a cumulative effect of iodine deficiency of the pre 2005 era. Most participants were women and most likely bore children during the period of iodine deficiency. This is similar to other studies where the ratio is skewed to women [14]. However enrollment of men was unexpectedly low, perhaps it could be explained by differences in health seeking behavior.

The impact of micronutrient deficiency is immense and such communities carry a heavy burden of it. The consequences of iodine deficiency include but not limited to goiter formation, thyroiditis and cretinism in children [19–21]. It may be worthwhile investigating iodine disorders among children and pregnant women in such communities and the barriers to control or elimination of iodine deficiency [21, 22].

The staple foods consumed were sorghum and maize. In the absence of iodized salt, they are unlikely to offer sufficient iodine intake [23]. The challenges of war and political upheaval cannot be under estimated in contributing to inadequate access to health care and lack of public health interventions including assessing micronutrient deficiency and the impact of interventions in place [9].

### Study limitations

Even though it was community based, it was not a house to house survey. We depended on participants responding to radio announcements. It is possible that many may not have turned up. However this method of mobilization is not new in the area, it has successfully been used before for child immunization campaigns.

Ultra-sonography has been used in epidemiological studies to assess thyroid size, leading to much higher estimates of goiter prevalence than in studies in which goiter size was assessed by physician examination [24]. Therefore it is likely that this study under estimates it’s prevalence in the population investigated.

In this study, we used urinary iodine excretion as a proxy to body iodine. It is a limitation in a way that it may not reflect iodine status of a participant in the past.

## Conclusion

In the population studied, the percentage of participants with median urinary iodine between 100-200 micro g/l indicating adequate intake and optimal nutrition was 52% leaving out nearly half of the population. Goiter was prevalent among the rural poor mostly women. Access to iodized salt for the rural poor in the underdeserved areas of south Sudan and sub Saharan Africa needs to be urgently improved.

## Abbreviations

IDDs: Iodine deficiency disorders
WHO: World Health Organization
UIC: Urinary iodine concentration.
